# Modeling Virus and Bacteria Populations in Europa’s Subsurface Ocean

**DOI:** 10.3390/life12050620

**Published:** 2022-04-21

**Authors:** Adriana C. Gomez-Buckley, Gordon M. Showalter, Michael L. Wong

**Affiliations:** 1 Department of Astronomy, University of Washington, Seattle, WA 98195, USA; mwong@carnegiescience.edu; 2 Astrobiology Program, University of Washington, Seattle, WA 98195, USA; gshowalt@uw.edu; 3 School of Oceanography, University of Washington, Seattle, WA 98195, USA; 4 NASA Nexus for Exoplanet System Science’s Virtual Planetary Laboratory, University of Washington, Seattle, WA 98195, USA; 5 Earth & Planets Laboratory, Carnegie Institution for Science, Washington, DC 20015, USA

**Keywords:** astrobiology, Europa, virus, bacteria, habitability

## Abstract

The search for life in the universe is often informed by the study of “extreme” environments on Earth, which provide analogs for habitable locations in the Solar System, and whose microbial inhabitants may therefore also serve as analogs for potential life forms in extraterrestrial milieus. Recent work has highlighted the ubiquity and importance of viral entities in terrestrial ecosystems, which calls for a greater understanding of the roles that viruses might play in hypothetical extraterrestrial biomes. While some studies have modeled the dynamics of viral and bacterial populations in icy ocean environments on Earth, previous work has yet to apply these findings to icy ocean worlds such as Jupiter’s moon Europa. It is commonly theorized that hydrothermal vents on Europa could produce the necessary reductants for chemosynthesis to take place on the ocean bottom. In the case that Europa’s ocean is a reductant-limited environment, how might reductants and organic matter reach the sub-ice region to power a more easily accessible ecosystem? Here, we propose a ‘viral elevator,’ a mechanism that functions similarly to the ‘viral shunt’ in Earth’s oceans, which could create and shuttle dissolved organic matter (DOM) to a hypothetical sub-ice biosphere through viral carriers. Current models of Europa’s ocean currents and stratification support the movement of DOM to the sub-ice biosphere. We adapt an existing model for bacterial and viral population dynamics in Earth’s Arctic sea ice to Europa and use parameters from various Arctic-based studies as proxies for Europa’s environment. We find that viral burst size has the most significant effect on the virus-to-bacteria ratio (VBR) and system longevity in closed systems (such as brine pockets within Europa’s icy crust), with higher burst sizes clearly increasing both. When applying our model to an open system with an influx of DOM from the viral elevator, we found that a steady-state system is attainable, with resulting sub-ice biofilms on the order of 0.1 mm thick (global equivalent layer). This has implications for future searches for life on Europa, given that life directly under the ice will be easier to detect and observe than life near the ocean bottom.

## 1. Introduction

On Earth, viruses that infect bacteria—known as bacteriophage viruses—play a key role in the cycling of marine organic carbon by releasing dissolved organic carbon and dissolved organic matter (DOM; <0.2 μm) through a process known as the ‘viral shunt’ [1]. Collins and Deming (2011) found “exceptionally high virus-to-bacteria ratios [VBR] in seawater (45–340) and sea ice (93–2820)” in the Amundsen Gulf region of the Arctic Sea, and concluded that polar sea ice on Earth may be a hotspot for virus–bacteria interactions [2]. Similarly, studies have demonstrated that microbial communities can thrive in extreme conditions—there is no cutoff of the metabolic rate even down to −40 °C [3,4], and lab studies have demonstrated microbial growth in permafrost as low as −15 °C [5]. Empirical studies have shown certain extremophile bacteria in biofilms to be culturable down to −25 °C [6].

Despite the prevalence of viruses on Earth, viruses have seldom been considered in astrobiology [7]. Recent studies have pointed out the critical role of viruses in the evolution of life on Earth and have pushed for further studies of viruses in other biospheres [8]. It has been hypothesized that in any biosphere, virus-like “genetic parasites” will naturally emerge [9], while others have speculated that viruses were instrumental in the origin and early evolution of cellular life itself [9,10,11,12]. Thus, if cellular life exists in Europa’s subsurface ocean, it is plausible that virus-like entities would also reside in that environment and play fundamental ecological roles. The high virus-to-bacteria ratio in terrestrial Arctic waters highlights the importance of viruses in Earth’s Arctic marine ecology, and implies that analogue environments, such as Europa, may also have viruses playing a key role in their global ecology.

Ever since the discovery of Europa’s global subsurface ocean [13,14], much work has gone into characterizing the habitability of this ocean for cellular life. Several studies have hypothesized that alkaline hydrothermal vents exist on Europa’s ocean bottom [15,16,17,18]. Based on the ecosystems that inhabit terrestrial submarine vents [19,20,21], sites such as these could provide favorable environments for extraterrestrial chemosynthetic life forms to thrive. Even without volcanic hydrothermal activity, studies have shown that reductants produced via serpentinization are still abundant enough to support life on a comparable scale to Earth’s oceans [22]. Heating from below and buoyant thermal plumes would cause ocean convection and currents that would transfer heat and other materials such as DOM to the liquid ocean’s surface, just under the ice [18]. Many studies have also focused on the viability of ice-derived oxidants, radiolytically generated at the surface of Europa, and subducted from the surface to the sub-ice region, possibly supporting microbial life on Europa [23,24,25,26].

## 2. The Viral Elevator Hypothesis

**Figure 1:**
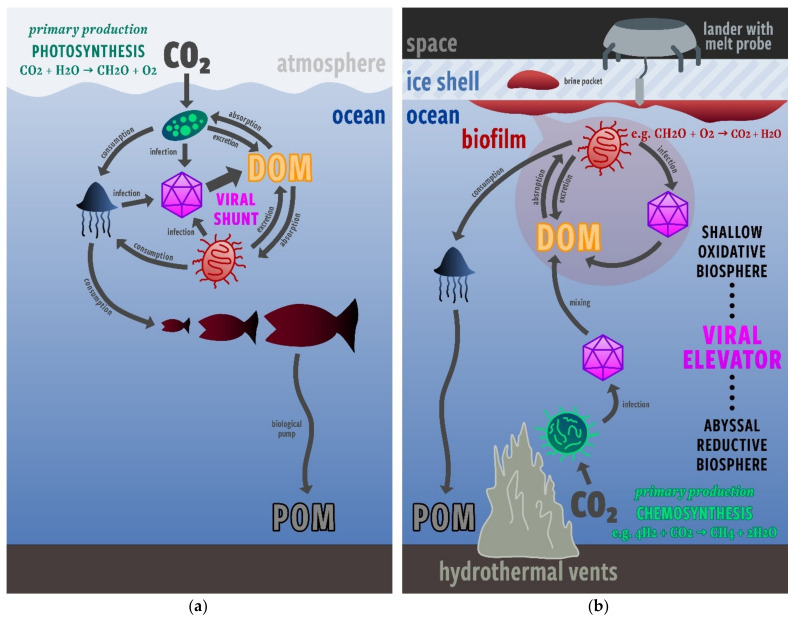
(**a**) A simplified model of the viral shunt in Earth’s oceans (after Breitbart et al., 2018) [1]. On Earth, carbon enters the system via photosynthesis at the top of the ocean, eventually settling at the bottom of the ocean as particulate organic matter (POM). The viral shunt assists in keeping organic matter in near-surface waters by creating dissolved organic matter (DOM) for further consumption. (**b**) A simplified model of the viral elevator in Europa’s ocean—a reverse of the viral shunt process on Earth. Here, carbon enters the system via chemosynthesis, fueled by reductants emitted at hydrothermal vents, at the bottom of the ocean. The viral elevator assists in creating DOM that can be transported from the benthic biosphere to a sub-ice biosphere, which would be much more accessible to near-future missions.

Here, we propose the possibility of a ‘viral elevator’—a process analogous to the viral shunt that allows viral activity to carry DOM from an abyssal, chemosynthetic, reductant-limited biosphere to a sub-ice, heterotrophic, DOM-limited biosphere (Figure 1). The viral elevator is the result of viral lysis in the abyssal biosphere, which liberates biological matter, fixed from CO_2_ by chemosynthetic microorganisms, into suspended DOM. This DOM is then transported upward through the ocean column via circulation and/or diffusion to the sub-ice biosphere, where it is able to be consumed by DOM-limited heterotrophs. This scenario rests on the assumption that Europa’s biosphere is net reductant limited, based on recent estimates showing that O_2_ fluxes from radiolysis could be comparable to or even overwhelm H_2_ fluxes from hydrothermal activity [22,27]. Hence, the viral elevator represents the primary mechanism for reduced organic matter to make it to the sub-ice biosphere, promoting the viability of this habitat.

The existence of a sub-ice biosphere would have positive implications for future missions to Europa searching for signs of life. The viral elevator would support active lifeforms that can be more easily observed just underneath the ice. A thriving sub-ice biosphere supported by a viral elevator would also increase the likelihood of biological material being entrained in putative Europan plumes, increasing the likelihood that biosignatures could be detected by a future Europa orbiter or lander. While it would still require drilling or melting through the ~10 km ice shell to observe Europan life in situ, it would be a much less complicated task than trying to find evidence of life that is confined to hydrothermal environments near the ocean bottom. The process of sending probes to the ocean bottom is already difficult on Earth, and would be much more complicated on Europa, whose ocean is ~1 order of magnitude deeper.

The state-of-the-art model of Europa’s ocean from Soderlund et al. (2019) shows that it is organized into large scale currents in three zonal jets and two equatorial circulation cells that are capable of transporting heat and materials between the ocean floor and surface [28]. We imagine the sub-ice biosphere to resemble either a shallow biosphere just beneath the ice, or perhaps biofilms growing on the underside of Europa’s ice cover [18], where prokaryotic analogs can receive an influx of oxidants from the convection of the ice above and an influx of DOM from the ‘viral elevator’ below. In sea-ice environments on Earth, biofilms have already been shown to play an important role in the flux of energy and matter [29].

Using a closed-system model for virus–bacteria dynamics in Earth’s sea ice [30], we used parameters from Arctic-based studies of bacterial and viral populations to simulate two different environments on Europa. Our closed-system model corresponds to a brine pocket within Europa’s ice shell and simulates the dynamics of viral and bacterial populations based on consumption of DOM from a fixed pool, with these populations eventually tapering off and dying out over time. By adding in a term for influx of DOM from our proposed viral elevator, we modeled an open system environment that corresponds to a sub-ice biosphere on Europa and found parameters that allow for a steady-state in the bacterial and viral populations.

## 3. Methods

### 3.1. Plausibility of the Viral Elevator

First, we perform an order-of-magnitude estimate, based on results from existing Europa literature, to confirm that the ‘viral elevator’ is plausible for Europa’s ocean through a combination of currents and diffusion. It is important to note that Soderlund’s models of energetic circulation prevent the formation of a substantial freshwater layer below Europa’s ice shell, meaning that DOM can be transported via convection from the abyssal to the sub-ice region of the ocean [28,31]. However, it is important to note that this study did not account for dissipation at the boundary layers of turbulent flows, which would decrease the velocity scale of oceanic flows and may not be adequate to penetrate a diffusive ceiling. Other viable models may also allow a freshwater layer to exist between the saline ocean and ice above in areas of low ocean turbulence [32]. This freshwater layer could disrupt the flow pattern of Europa’s ocean, acting as a stably stratified ‘lid’—therefore, DOM would have to depend on diffusion to move through this layer in certain areas. We take the diffusion constant value $C_{diff}$ to be approximately $0.5\times 10^{-9}\frac{m^{2}}{s}$, based on values for organic compounds in water [33]. Zhu et al. (2017) calculate the freshwater layer to be between $d_{min}=107\;m$ and $d_{max}=8000\;m$ [32]. Based on these values, we find that the timescale for DOM diffusion through the freshwater layer $\tau_{diff}$ to be between ~$7\times 10^{5}$ and $4\times 10^{9}$ yr. Given that Kalousova et al. (2014) give the timescale for Europa’s ice sheet convection to be between ~1 and 100 kyr [34], the diffusion of DOM through the freshwater layer can occur on a similar timescale so long as the freshwater layer is no thicker than ~800 m. If so, then DOM can viably be shuttled to the surface through a combination of currents and diffusion.

### 3.2. Model and Parameters

Our closed-system version of the single-species model uses a simple set of three differential equations describing the time evolution of DOM per unit volume (*N*), bacteria per unit volume (*B*), and viruses per unit volume (*V*), shown below:

(1) $$\frac{dN}{dt}=\;-\alpha \left(\frac{N}{N+Q}\right)B+g\left[\alpha \left(\frac{N}{N+Q}\right)B\right]+\left[n\gamma \phi VB\cdot \left(1\times 10^{-7}\right)\right],$$

(2) $$\frac{dB}{dt}=\mu \left(\frac{N}{Q+N}\right)B-\phi VB-d\cdot B,$$

(3) $$\frac{dV}{dt}=\gamma \beta \phi VB-\phi VB-mV,$$

with parameters and values listed in Table 1.

The open-system version augments the first equation with the additional term *E*. Physically, *E* represents the flux of DOM generated by viral lysis in the benthic biosphere and transported to the sub-ice biosphere that sustains the open system and has units of $\frac{\mu g}{\mathrm{mL}\cdot \mathrm{hr}}$. *E* essentially replaces the DOM lost to bacterial death other than by viral lysis, which results in the production of particulate organic matter (POM) that sequesters out of the sub-ice biosphere system. The *E* required to sustain the open system can be estimated taking the time-average of $f\left(\frac{\alpha }{\mu }\right)d\cdot B$, because $\frac{\alpha }{\mu }$ represents the conversion factor between DOM and a bacterial cell ($\frac{\mu g}{\mathrm{cell}}$) and d⋅B is the is rate of bacterial death not due to viral activity ($\frac{\mathrm{cell}}{\mathrm{mL}\cdot \mathrm{hr}}$). We find empirically that a factor of *f*~70–85 results in long-lived open system biospheres with steady DOM concentrations (*f* ≲ 70 results in biospheres that lose viability before 200,000 h; *f* ≳ 85 results in infinitely increasing DOM concentrations).

We based our parameters on studies of Arctic bacterial and viral populations, as proxies for Europa conditions, to solve the system of equations and model the relationship between bacterial population, viral population, and amount of DOM in the system (Table 1). Below, we describe our choices for our model parameters and initial conditions.

To account for our uncertainty with regard to a Europan biosphere, we initialized each model run with a random concentration of bacteria (10^4^–10^6^ mL^−1^) and viruses (10^5^–10^7^ mL^−1^). Our bacterial values are based on the results of Nguyen and Maranger (2011), who studied bacterial production and respiration rates in the sea ice and underlying waters around Banks Island (Northern Canada) [35]. They found a bacterial abundance of $2.99\times 10^{5}$ cells mL^−1^ in the ice, and an abundance of $2.27\times 10^{5}$ cells mL^−1^ in the ice-water interface. Typically, in seawater, VBRs can range between 1 and 100 viruses per bacterium [30].

Furthermore, we initialized each model run with a random temperature and bacterial growth rate. Our temperature parameter (*T*) ranges from –4 to 0 °C based on the model of Europa’s ocean temperatures from Melosh et al. (2004) [36]. The uptake constant (α) is scaled using the *Q*_10_ law based on this temperature range, and both α and the half-saturation constant *Q* are taken from Showalter (2020). Likewise, the bacterial growth rate (μ) was averaged from the range of values according to Showalter (2020) and varied ± the geometric mean of the range [30].

The constant of bacterial death (*d*) is taken from a study done by Servais et al. (1985) of bacterial populations in Belgian coastal waters, and scaled using the *Q*_10_ law for Arctic Ocean temperatures [37].

The fraction of uptake material recycled into the nutrient pool as exudate (*g*) was taken according to Marx et al. (2009) to be 0.2 [38]. As this parameter is not well constrained for Earth’s oceans, we chose a range starting one order of magnitude lower of 0.02–0.2 to test values between a low and high exudate. The fraction of viral lysis material recycling into nutrient pool (*n*) was taken according to Jover et al. (2014) [39].

Our constant of viral decay was found by using a decay rate given in Dell’Anno et al. (2015), who conducted a study of viral decay rates in deep sea sediments around the Svalbard archipelago (Norway) [40]. Given that viruses in Europa’s sub-ice biosphere would not be affected by UV stress like those on Earth, deep sea sediments were chosen as an analogue rather than a sub-ice location. Using their given decay rate of $5.26\times 10^{12}\frac{\mathrm{cells}}{m^{2}\cdot d}$ and their given viral abundance for the Arctic Ocean, $V≃1.5\times 10^{9}\frac{\mathrm{cells}}{g}$, we assumed a measurement sample depth of 1 cm for their given 10^4^ cm^3^ sample field, and calculated m=0.015 h^−1^.

**Table 1:** Parameters.

Parameter	Units	Description	Europa-Proxy Value *	Source
$N_{i}$	$\frac{\mu g}{\mathrm{mL}}$	Initial amount of DOM (nutrient pool)	10−50	[30]
$B_{i}$	$\frac{\mathrm{cells}}{\mathrm{mL}}$	Initial bacterial population	$1\times 10^{4}-1\times 10^{6}$	[35]
$V_{i}$	$\frac{\mathrm{cells}}{\mathrm{mL}}$	Initial viral population	$1\times 10^{5}-1\times 10^{7}$	[30,35]
T	C	Temperature	−4−0	[36]
α **	$\frac{\mu g}{\mathrm{cell}\cdot \mathrm{hr}}$	Uptake constant	$\left(1.2\times 10^{-7}\right)\times \left(3^{\frac{T-23}{10}}\right)$	[30]
μ	${\mathrm{hr}}^{-1}$	Bacterial growth rate	0.016−0.032	[30]
Q	$\frac{\mu g}{\mathrm{mL}}$	Half-saturation constant	0.022	[30]
d	${\mathrm{hr}}^{-1}$	Constant of bacterial death	0.0002	[37]
m	${\mathrm{hr}}^{-1}$	Viral decay rate	0.015	[40]
g	−	Fraction of uptake material recycled into nutrient pool as exudate	0.02–0.2	[38]
n	−	Fraction of viral lysis material recycling into nutrient pool	0.99	[39]
γ ***	−	Lytic vs. lysogenic fraction	0−1	[30]
ϕ	$\frac{\mathrm{mL}}{\mathrm{cell}\cdot \mathrm{hr}}$	Adsorption (infection) rate	$1\times 10^{-11}-1\times 10^{-9}$	[41]
β	$\frac{\mathrm{cells}}{\left[\mathrm{burst}\right]\;\mathrm{cell}}$	Viral burst size	10−500	[41]
f	$\frac{\mu g}{\mathrm{cell}}$	Conversion rate between grazed bacteria and DOM	70−85	This study

* Values here are based on studies of Arctic bacterial and viral populations which we use as proxies for conditions on Europa. All parameters listed with respective units. Ranges indicate random seed values for parameters which are not well constrained. ** α is scaled according to the Q_10_ law. *** γ ranges between fully lytic (0) and fully lysogenic (1).

Vaque et al. (2019) studied bacterial production and mortality, as well as viral lysogenic activity, in Svalbard Fjord (Norway) [41]. The percentage of bacteria lysed by viruses (*L_B_*) was 0.07 d^−1^, with a viral concentration (*V_C_*) of approximately $2\times 10^{7}\frac{\mathrm{cells}}{\mathrm{mL}}$. From these values we calculated an infection rate ϕ of:

(4) $$\phi =\frac{L_{B}}{V_{C}}=1\times 10^{-10}\frac{\mathrm{mL}}{\mathrm{cell}\cdot \mathrm{hr}}\;,$$

To account for our uncertainty with regard to virus–bacteria affinities in an extraterrestrial ecosystem, we set the infection rate ϕ in each run to a random value taken from a range of 10^–11^–10^–9^ $\frac{\mathrm{mL}}{\mathrm{cell}\cdot \mathrm{hr}}$. 

For our last parameter, burst size, we also considered a range of values. Vaque et al. (2019) stated that in Arctic waters, viral burst size could range from 9 to 500 viruses per bacterium [41]. Thus, we tested arbitrary values in our model such that our final five burst size values, listed below, adequately show the model’s behavior and its changes from the lower to the upper limit.

## 4. Results

### 4.1. Closed System

The closed-system model corresponds to the case of a viral–bacterial ecosystem closed off from an influx of DOM, such as in a brine pocket within Europa’s ice shell. DOM is consumed by bacteria and liberated by viral lysis, oscillating in response to peaks of microbial activity, but showing a steady overall decrease with time as the initial DOM pool is consumed.

**Figure 2:**
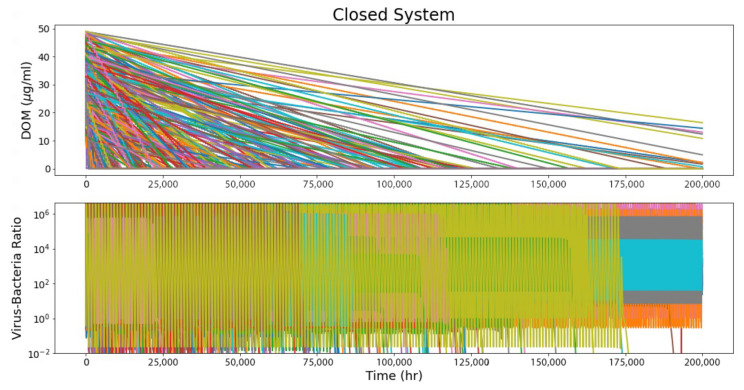
Closed-system model results from 300 runs (with 31 outliers removed). Each color represents an individual run. Parameters were randomly seeded where applicable (Table 1), resulting in a wide range of system longevity and VBR ranging between ~10^−2^–10^6^. All modeled systems trend to zero (i.e., the finite DOM supply is consumed and the system loses viability).

Figure 2 shows the behavior of the system parameters over time. In order from top to bottom: the amount of DOM in the system, the bacterial population (*B*), the viral population (*V*), and the VBR. Each color represents a separate run with randomly seeded parameters where applicable (see Table 1).

**Figure 3:**
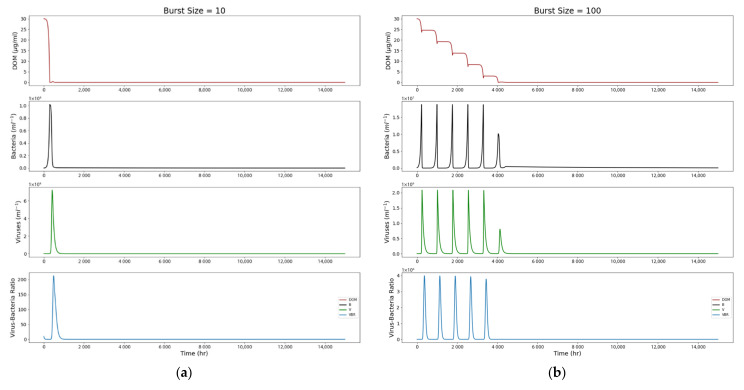

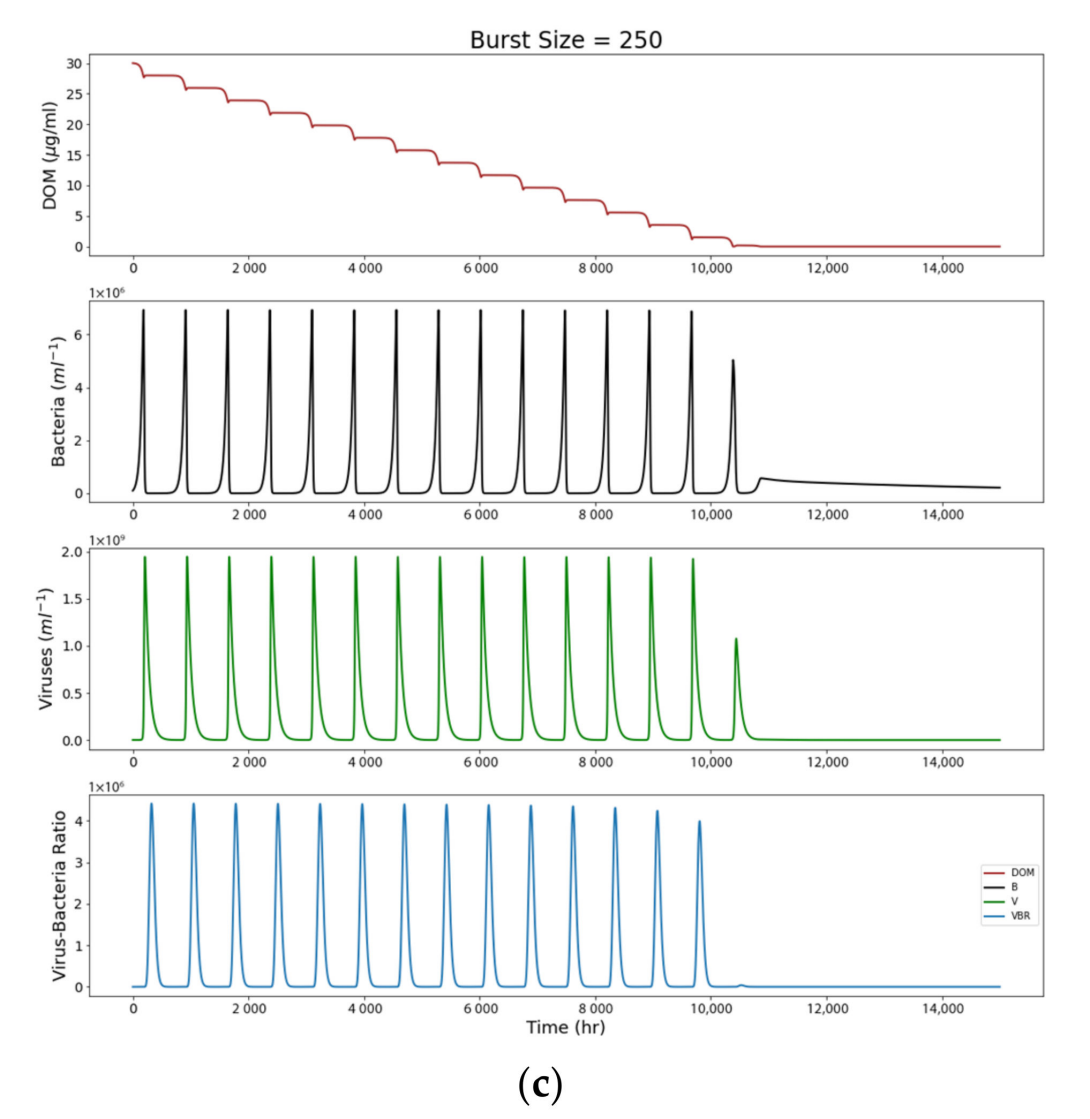
Closed-system model results from a single run over 15,000 h, with varying viral burst sizes to show differences in system longevity. Higher viral burst sizes result in greater system longevity. (**a**) Burst size 10; (**b**) burst size 100; (**c**) burst size 250.

To better observe the behavior of each parameter, Figure 3 shows a single run of the model over 15,000 h with set starting values as follows: DOM = 30; *B* = 10^5^; *V* = 10^6^; T=−2; g=0.09; μ = 0.024; γ=1; ϕ = 10^−10^. The burst size β has values of 10, 100, and 250, labeled accordingly.

We observe the bacterial population increasing as the DOM is consumed, and with a slight time delay, the viral population begins to increase as the bacterial population peaks. As bacteria are lysed, *B* decreases. *V* peaks and then decreases as the bacterial population is depleted, leaving few bacteria for the viruses to infect. Once the viral population is significantly diminished, the cycle repeats. The fourth plot shows us the VBR over time. As this is a closed system, the overall trend shows that these interactions eventually taper off to zero over time.

**Figure 4:**
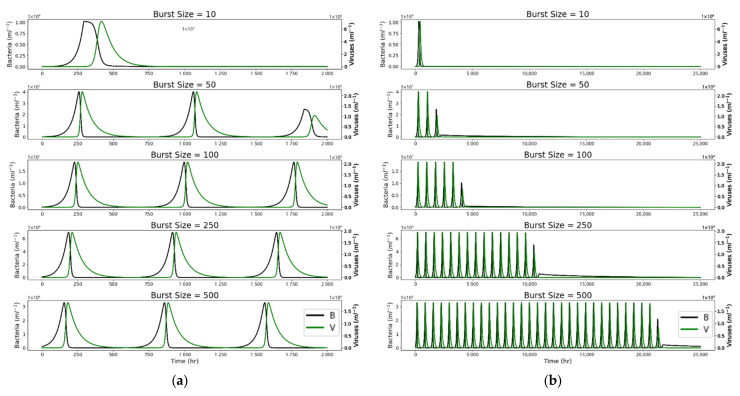
(**a**) Bacterial and viral populations with varying burst size over 2000 h. Time between peaks slightly decreases as viral burst size increases, however, for a maximum viral burst size of 500, the time between peaks is still significant (~28 days). (**b**) Bacterial and viral populations with varying burst size over 25,000 h. For a maximum viral burst size of 500, populations persist for ~3 years.

Figure 4 uses the starting values from Figure 2 and compares *V*(*t*) and *B*(*t*) over time for runs with various burst sizes, labeled accordingly. Figure 4a compares these populations over the first 2000 h. Figure 4b compares these populations over 25,000 h.

Figure 4a shows that larger burst sizes result in a higher frequency of population peaks. However, even for a maximum burst size of 500, the time between population peaks is still ~28 days. Additionally, the bacterial population varies between a minimum of ~60 mL^−1^ and a maximum of ~1 × 10^8^ mL^−1^. The viral population varies even more, between a minimum of ~0.1 mL^−1^ and a maximum of 2 × 10^9^ mL^−1^. The bacterial population at its minimum is therefore a mere 6 × 10^−7^ of the population at its peak, while the viral population minimum is 5 × 10^−11^ of its maximum. This has interesting implications for the search for life on icy ocean worlds. We can use the wildflower blooms of Death Valley as an analogy: if one were to visit and observe for a short period in between blooms, it would seem that the area is devoid of flowering plants, whereas observation over a longer time would show this not to be the case. While bacterial and viral population peaks in our model of Europa’s ocean are functionally different from wildflower blooms, the implications are similar—that observing during a short period between population peaks could result in a false negative for the presence of life. These results suggest that mission longevity and continuous measurements could be important to the success of future life detection missions to icy ocean worlds.

Figure 4b reveals another interesting result—a larger burst size increases the overall longevity of the bacterial–viral system, causing it to taper off at a much later time. Here, the viruses appear to act as a “population control” for the bacterial population. In a system with a burst size of only 10 viruses per lysed bacterium (top plot of Figure 4b) shows that the bacterial population spikes without viral interference; corresponding plots show that the DOM pool is quickly depleted (Figure 3). Thus, the populations only peak once. However, a burst size of 500 allows the populations to persist until ~25,000 h. The more robust viral population prevents the runaway growth of the bacterial population and the subsequent depletion of the DOM pool; viral lysis also recycles some DOM back into the pool to be available for future generations of bacterial hosts. This can be seen in the much more gradual decline of DOM in the corresponding plot in Figure 3.

Our closed-system model conditions are relevant to microbial populations existing in places such as a brine pocket within the ice. For a maximum burst size of 500, the system would persist for a maximum of ~3 years. Given that the timescale for convection of ice on Europa is ~1–100 kyr, microbial life in a brine pocket would not persist long enough to reach the surface of Europa alive [34]. Therefore, while surface landers could theoretically observe dead organisms or remnants of biological organic material, the ideal place to look for active microbial populations would be in a location with open-system conditions, such as the sub-ice ocean.

### 4.2. Open System

**Figure 5:**
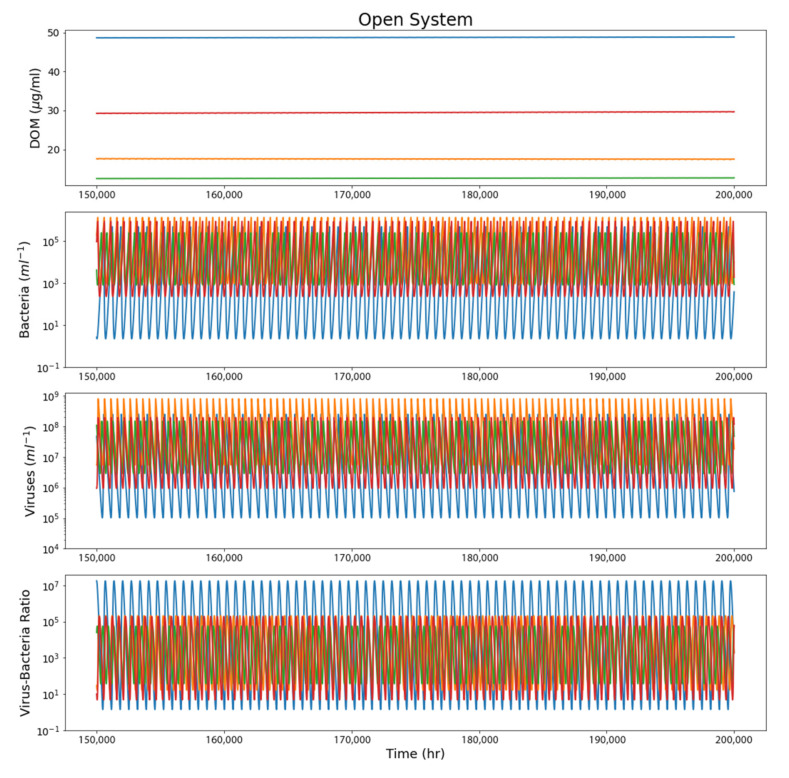
A sample of 4 runs from the open-system model to better demonstrate the behavior of the steady state. Plots show the last 50,000 h (2500 runs in total were done, with 2342 outliers removed). These runs clearly demonstrate a steady state with no significant increase or decrease in each parameter over time.

The open-system model corresponds to a case where a viral–bacterial ecosystem is constantly supplied with DOM from the viral elevator, such as a sub-ice biosphere. As in the closed-system case, the DOM oscillates with microbial activity, but steady-state systems could be achieved over long timescales. Steady state cases were defined as model runs in which the overall DOM concentration did not change appreciably over the entire model run (DOM values were averaged over the first and last 20,000 h of the model run; runs with an increase or decrease of more than 10% were classified as non–steady state and removed) and the bacteria and virus populations continued to oscillate about a steady value without completely dying out (Figure 5).

**Figure 6:**
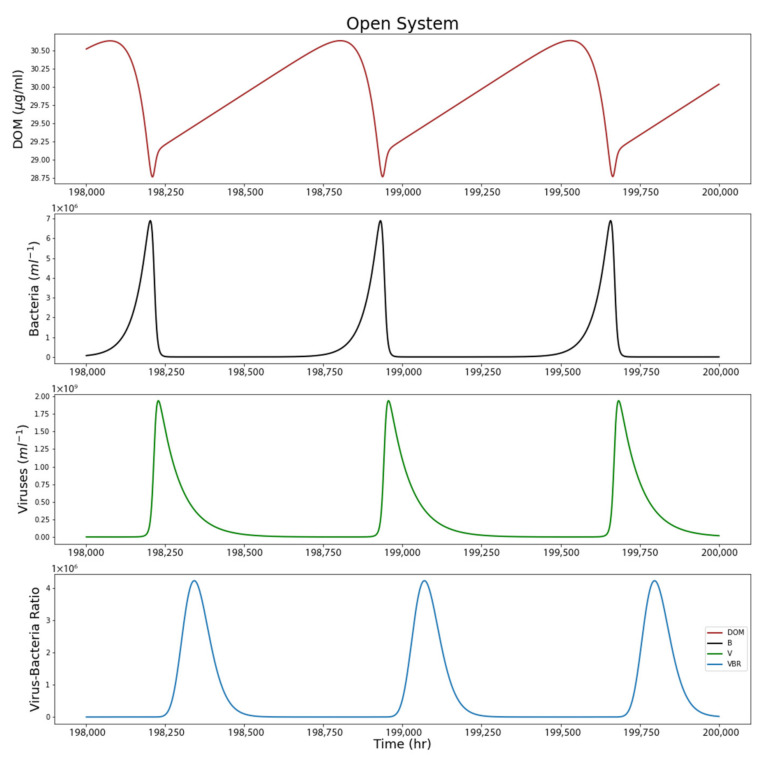
Open system model results showing the last 2000 h for a steady state system. Unlike the closed-system model, open-system runs do not taper off to zero over time.

Figure 6 shows a closer look at the nature of the steady state, with set starting values from Figure 3. The resulting steady state value for *f* was 72.416.

**Figure 7:**
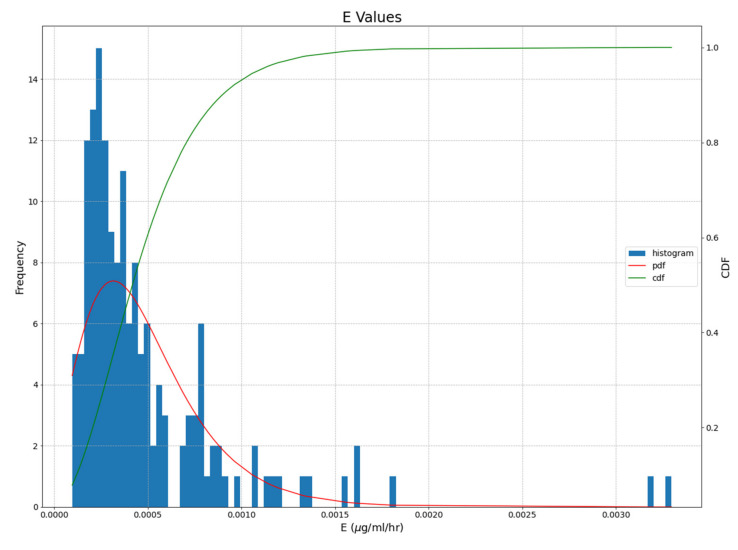
Histogram of average E values with log-normal distribution curve (pdf) and cumulative distribution function curve (cdf) fitted to the model outputs. Based on the cdf, it is 90% likely that E is less than ~1.2 × 10^−3^ but above the lower limit of ~1 × 10^−4^.

*E* values were averaged over each run and compiled into a histogram fitted with a log-normal distribution curve (Figure 7).

VBR values were also averaged over each run. The median VBR was 4.7 × 10^4^, with lower and upper quartiles at 891 and 5.8 × 10^5^, respectively. The majority of VBR values were around 100 or less. This is in good agreement with terrestrial VBR values for the Arctic [2]. Given that in Earth’s oceans the VBR is ~10 and yet viruses only make up ~5% of the prokaryotic biomass, a VBR of ~200 will result in a roughly equal split of biomass between viruses and bacteria [42]. Our models suggest that for reasonable burst sizes, viruses on icy worlds could outweigh their bacterial hosts in biomass. Thus, our search for life on Europa and other icy ocean worlds should certainly account for viruses. Not only would they be highly abundant, but given the slight time delay in the peak of the viral population after the peak of bacterial population, a bacteria-based search for life could struggle to find positive readings during times where the viral population would still be blooming.

We assume that the DOM that fuels the sub-ice biosphere ultimately comes from inorganic carbon fixed by life in the benthic biosphere, liberated into DOM by viral activity, and transported upwards through the ocean column via the viral elevator (Figure 1). Thus, to constrain the possible depth of a sub-ice biosphere on Europa, we first estimated the benthic biomass synthesis rate ($B_{R}$, in $\frac{\mathrm{kg}}{\mathrm{yr}}$) on Europa using the following equation:

(5) $$B_{R}=r\times G\times c\times 10^{-3},$$

where r is the geochemical reductant (H_2_) flux in $\frac{\mathrm{mol}}{\mathrm{yr}}$, G is the Gibbs free energy for the given metabolic redox reaction in $\frac{\mathrm{kJ}}{\mathrm{mol}}$, and c is the anabolic conversion factor in grams of biomass per kJ of free energy. Vance et al. (2016) gives the flux of H_2_ from water-rock reactions to be $10^{10}\frac{\mathrm{mol}}{\mathrm{yr}}$ and show that this H_2_ flux is likely matched or exceeded by the O_2_ flux into Europa’s ocean from surface ice radiolysis [22]. Hence, the dominant geochemical gradient at the Europan seafloor can be considered to be one in which H_2_ serves as the reductant and O_2_ serves as the oxidant. The Gibbs free energy for H_2_ reacting with O_2_ is $237.13\;\frac{\mathrm{kJ}}{\mathrm{mol}}.$ Taking the conversion factor $c=\frac{1\;g}{4.1868\;\mathrm{kJ}}$ from Chyba et al. (2001), we get $B_{R}\sim \;6\times 10^{8}\frac{\mathrm{kg}}{\mathrm{yr}}$ [15].

The viral elevator input rate *E* required to sustain a sub-ice biosphere with a certain thickness given a plausible benthic biomass synthesis rate can be calculated via the following equation:

(6) $$E=\frac{\frac{B_{R}}{b_{m}}\times \frac{n}{8760}}{\left(4\pi r^{2}\right)\times \left(d\times 10^{-3}\right)\times 10^{6}},$$

where $b_{m}$ is the mass of a bacterium (10^−15^ kg), n is the amount of DOM ($6\times 10^{-8}\frac{\mu g}{\mathrm{cell}}$), r is the radius of Europa ($1.5\times 10^{6}m$), and d is the depth of the sub-ice biosphere in mm. The numerator gives a biomass synthesis rate in $\frac{\mu g}{\mathrm{hr}}$, while the denominator gives a volume in ml, which results in our viral elevator input rate with units $\frac{\mu g}{\mathrm{mL}\cdot \mathrm{hr}}$.

**Figure 8:**
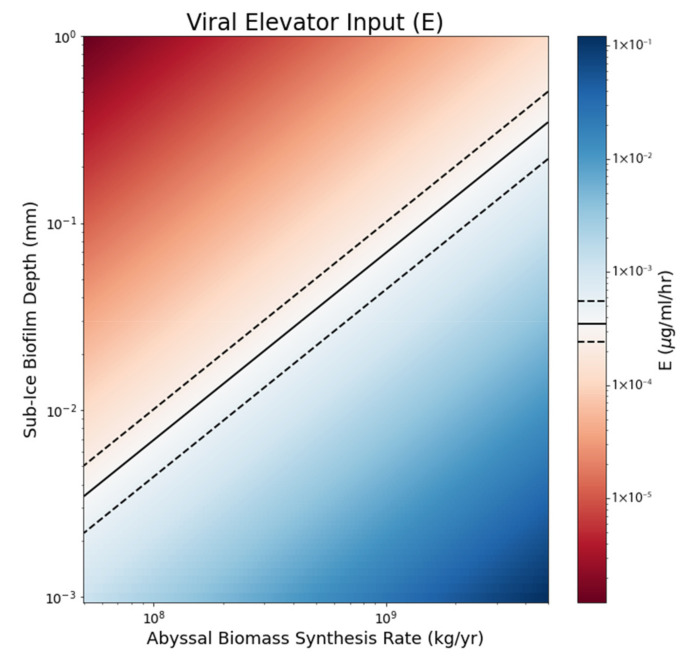
Colormap diagram of abyssal biomass synthesis rate vs. depth of sub-ice biofilm. Solid contours show the median of the averaged E values from Figure 7, while dashed contours represent the interquartile range. Values for biomass and depth that fall within the blue support a steady state system, while values falling in the red do not. Values that support a steady state suggest that a sub-ice biofilm would be no more than 0.1 mm in thickness.

We vary the benthic biomass synthesis rate by an order of magnitude in either direction of the estimate from above (B_R_~6 × 10^8^ kg/yr) and calculate the viral elevator flux *E* required to produce sub-ice biofilms of various depths (Figure 8). We overlay the median and interquartile values of the averaged E values from our simulations to indicate the most plausible biofilm depth for a given benthic biomass synthesis rate.

As previously discussed, life under the ice on Europa may take the form of biofilms—thus, our colormap suggests that this biofilm would be no more than 0.1 mm in thickness, in terms of a global equivalent layer (GEL). For comparison, studies of microbial biofilms in Earth’s Arctic regions have shown that these mats can be up to several mm thick [43,44]. Other controlled studies of biofilms have shown that they can range from 5–1000 µm thick, with mean thicknesses of 200–400 µm [45,46,47]. However, such terrestrial microbial mats are not directly analogous given that these mats are in locations with shallower depths and thinner ice which allows photosynthetic organisms to survive. Thus, it is reasonable that on Europa, where the thick ice prevents photosynthesis, purely chemosynthetic biofilms would be thinner than those found on Earth.

## 5. Discussion

Our findings show that viruses could play an ecologically important role in a hypothetical biosphere on Europa by imparting homeostatic regulation through bacterial lysis and the recycling of DOM. Viral activity on Europa could prolong the viability of an ecosystem in brine pockets and sustain a heterotrophic sub-ice biosphere for the long term, similar to how the viral shunt helps maintain marine ecosystems on Earth. These findings are consistent with the idea that ecosystem-level homeostasis is a pillar of living systems in general [48], and further motivates the idea that viruses should be accounted for in astrobiological studies [7]. 

Still, much work remains to characterize Europa’s habitability and model the full nature of a co-evolving system of virus and bacteria analogs in extraterrestrial environments. To assess the viability of the viral elevator concept, we use models of Europa’s ocean from Soderlund et al. (2014) and Soderlund (2019) [28,31]. These models have energetic circulation which would prevent the formation of a significant sub-ice freshwater layer—however, as specified in Zhu et al. (2017), there are other studies which propose models with weaker circulation regimes, and would allow the existence of this freshwater layer [32]. A large enough layer of freshwater would impede DOM movement on a reasonable timescale to the sub-ice biosphere.

Additionally, much is still unknown about the conditions of the ocean beneath the ice, and our given parameters that come from an Arctic-Ocean environment may be incorrect. As discussed previously, the closed system model is relevant to limited locations, such as brine pockets within the ice. While the results of our closed-system model already show that there are significant periods (~28 days) of low bacterial and viral populations, we also acknowledge that in a more DOM-limited environment these populations may lie dormant (e.g., in sporulated form for bacteria, changing lytic vs. lysogenic state for viruses) for long periods of time.

Our open system model is limited in that it does not account for a DOM influx from sources other than the viral elevator (such as carbon reduction at hydrothermal systems and the subduction of surface organics into the ocean) and assumes a DOM-limited or reductant-driven sub-ice ecosystem. Other studies have assumed an oxidant-limited or oxidant-driven system, where radiation on the surface of Europa creates oxygen and hydrogen peroxide that are subducted and eventually reach the sub-ice biosphere [15,16,17]. Estimates from these studies predict enough oxidants to support macrofauna similar to that found in Earth’s ocean in zones with minimal oxidants [16,17]. However, Vance et al. (2016) show that the flux of oxygen to Europa’s ocean would likely dominate over the flux of reductants [22]. While it is possible that such systems on Europa may be CO_2_-limited, we suspect that this is not the case given that CO_2_ is the most abundant gas in plumes on Enceladus, a similar icy moon to Europa [49]. Regardless, we acknowledge that our open system model is a simplified representation of Europa’s biogeochemical cycles, given the likely flux of reductants from the surface.

It is also possible that marine systems on Europa are limited by some other nutrient besides DOM, just as photosynthetic marine systems on Earth are limited by nitrogen, phosphorus, and/or iron [50,51,52]. Currently, elemental concentrations in Europa’s ocean are not well known, and the exobiological requirements of certain trace nutrients are highly speculative, so we leave it to future studies to investigate the implications of such scenarios.

It should also be noted that our model does not account for a few important biological factors. The first of these factors is the speciation of bacteria or viruses. While it is currently impossible to know what the speciation on Europa would look like, we can still model this behavior off of an Arctic-like icy ocean environment. A future version of our model might include different bacterial genotypes and respective phages, and allow for evolution, similar to the work of Beckett and Williams (2013) [53]. Having many different species of bacteria and viruses may result in many smaller population peaks at slightly different times, which would likely smooth out the “boom–bust” cycles we observe in our simple system.

A secondary factor is temperature dependence, which is limited in our model. Currently, the only parameter affected by temperature is the uptake constant α, which is scaled according to the *Q*_10_ law. However, especially in the case of our closed system model, several parameters are likely affected by temperature, given that temperature affects the relative contact rate (RCR) between bacteria and viruses. Future iterations of this model may include initial values for DOM, bacteria, and viruses as well as viral burst size (β), bacterial growth rate (μ), and infection rate (ϕ) as a function of temperature. Future work may also take into account sporulation and changes to the lytic vs. lysogenic state by making bacterial growth rate (μ), uptake constant (α), and lytic vs. lysogenic fraction $\left(\gamma \right)$ functions of DOM availability and/or other parameters of the model.

Lastly, it should be noted that the parameters *g* and *n* (Table 1) are not well constrained for Earth’s oceans, and may affect the accuracy of our model results. With future studies that further constrain these parameters in Arctic ocean environments, our analogs for Europa can also be improved.

## 6. Conclusions

In this paper, we developed a model of virus–bacteria population dynamics for hypothetical biospheres on icy ocean worlds and applied it to the case of Europa’s subsurface ocean, modeling two different scenarios: a closed system that has a finite supply of DOM and an open system that feeds off of a continuous supply of DOM. We introduced the novel concept of a ‘viral elevator’ that could hypothetically transport DOM in a reductant-limited system from the bottom of the ocean to the top of the ocean, fueling sub-ice biosphere that would persist indefinitely underneath the ice, perhaps as a biofilm.

Using Arctic Ocean-based parameters as proxies for the environment on Europa, we have found an interesting relation between viral burst size and the longevity of our closed system’s bacterial and viral populations. A more robust viral population appears to keep the bacterial population in check, allowing both populations to persist longer. There were long periods of time (~28 days) between bacterial and viral population peaks; overall the populations persisted for a maximum of ~25,000 h. Given timescale estimates for ice convection on Europa, the equivalent of such a system—say, in a brine pocket within the ice—would not persist long enough to reach the surface alive. Thus, searches for life would need to penetrate Europa’s icy shell and reach an open system environment to observe active microbial populations.

When an influx of DOM from the viral elevator was applied to model an open system, it resulted in a steady state. VBR values here were in good agreement with terrestrial marine VBRs, and viruses constituted a large fraction of the system’s biomass. By calculating the biomass synthesis rate based on reductant flux estimates from previous studies, we constrained the possible depth of a sub-ice biosphere on Europa to be on the order of ~10^−3^–10^−1^ mm GEL.

The overall abundance of viruses and significant time between population peaks suggest that future probes for life on Europa and other icy ocean worlds should include viruses in their search rather than only prokaryotic analogs, and that observing and sampling for life should span several weeks. Probes should focus on the sub-ice biosphere, where the presence of a viral elevator on Europa would likely mean the presence of a stable, active microbial community. The existence of a viral elevator would make the search for life on Europa more promising by bringing life within closer reach of future landers and probes.

## Data Availability

Not applicable.

## References

[1] Breitbart M., Bonnain C., Malki K., Sawaya N.A. (2018). Phage puppet masters of the marine microbial realm. Nat. Microbiol..

[2] Collins R.E., Deming J.W. (2011). Abundant dissolved genetic material in Arctic Sea ice Part II: Viral dynamics during autumn freeze-up. Polar Biol..

[3] Price P.B., Sowers T. (2004). Temperature dependence of metabolic rates for microbial growth, maintenance, and survival. Proc. Natl. Acad. Sci. USA.

[4] Merino N., Aronson H.S., Bojanova D.P., Feyhl-Buska J., Wong M.L., Zhang S., Giovannelli D. (2019). Living at the extremes: Extremophiles and the limits of life in a planetary context. Front. Microbiol..

[5] Mykytczuk N.C.S., Foote S.J., Omelon C.R., Southam G., Greer C.W., Whyte L.G. (2013). Bacterial growth at −15 °C; molecular insights from the permafrost bacterium *Planococcus halocryophilus* Or1. ISME J..

[6] Frösler J., Panitz C., Wingender J., Flemming H.C., Rettberg P. (2017). Survival of *Deinococcus geothermalis* in biofilms under desiccation and simulated space and martian conditions. Astrobiology.

[7] Berliner A.J., Mochizuki T., Stedman K.M. (2018). Astrovirology: Viruses at Large in the Universe. Astrobiology.

[8] Trubl G., Stedman K., Bywaters K., Boston P.J., Kaelber J.T., Roux S., Rodríguez-Román E. (2021). Astrovirology: Expanding the Search for Life. Bull. Am. Astron. Soc..

[9] Durzyńska J., Goździcka-Józefiak A. (2015). Viruses and cells intertwined since the dawn of evolution. Virol. J..

[10] Forterre P. (2006). The origin of viruses and their possible roles in major evolutionary transitions. Virus Res..

[11] Forterre P., Prangishvili D. (2009). The great billion-year war between ribosome-and capsid-encoding organisms (cells and viruses) as the major source of evolutionary novelties. Ann. N. Y. Acad. Sci..

[12] Krupovic M., Dolja V.V., Koonin E.V. (2019). Origin of viruses: Primordial replicators recruiting capsids from hosts. Nat. Rev. Genet..

[13] Kivelson M.G., Khurana K.K., Joy S., Russell C.T., Southwood D.J., Walker R.J., Polanskey C. (1997). Europa’s magnetic signature: Report from Galileo’s pass on 19 December 1996. Science.

[14] Kivelson M.G., Khurana K.K., Russell C.T., Volwerk M., Walker R.J., Zimmer C. (2000). Galileo Magnetometer Measurements: A Stronger Case for a Subsurface Ocean at Europa. Science.

[15] Chyba C.F., Phillips C.B. (2001). Possible ecosystems and the search for life on Europa. Proc. Natl. Acad. Sci. USA.

[16] Hand K.P., Carlson R.W., Chyba C.F. (2007). Energy, Chemical Disequilibrium, and Geological Constraints on Europa. Astrobiology.

[17] Greenberg R. (2010). Transport rates of radiolytic substances into Europa’s ocean: Implications for the potential origin and maintenance of life. Astrobiology.

[18] Russell M.J., Murray A.E., Hand K.P. (2017). The Possible Emergence of Life and Differentiation of a Shallow Biosphere on Irradiated Icy Worlds: The Example of Europa. Astrobiology.

[19] Kelley D.S., Karson J.A., Früh-Green G.L., Yoerger D.R., Shank T.M., Butterfield D.A., Hayes J.M., Schrenk M.O., Olson E.J., Proskurowski G. (2005). A Serpentinite-Hosted Ecosystem: The Lost City Hydrothermal Field. Science.

[20] Brazelton W.J., Schrenk M.O., Kelley D.S., Baross J.A. (2006). Methane- and Sulfur-Metabolizing Microbial Communities Dominate the Lost City Hydrothermal Field Ecosystem. Appl. Environ. Microbiol..

[21] López-García P., Vereshchaka A., Moreira D. (2006). Eukaryotic diversity associated with carbonates and fluid—Seawater interface in Lost City hydrothermal field. Environ. Microbiol..

[22] Vance S.D., Hand K.P., Pappalardo R.T. (2016). Geophysical controls of chemical disequilibria in Europa. Geophys. Res. Lett..

[23] Hand K.P., Carlson R.W. (2011). H_2_O_2_ production by high-energy electrons on icy satellites as a function of surface temperature and electron flux. Icarus.

[24] Teolis B.D., Plainaki C., Cassidy T.A., Raut U. (2017). Water Ice Radiolytic O_2_, H_2_, and H_2_ O_2_ Yields for Any Projectile Species, Energy, or Temperature: A Model for Icy Astrophysical Bodies. J. Geophys. Res. Planets.

[25] Galli A., Vorburger A., Wurz P., Pommerol A., Cerubini R., Jost B., Poch O., Tulej M., Thomas N. (2018). 0.2 to 10 keV electrons interacting with water ice: Radiolysis, sputtering, and sublimation. Planet. Space Sci..

[26] Li J., Gudipati M.S., Mishra Y.N., Liang M.-C., Yung Y.L. (2021). Oxidant generation in the ice under electron irradiation: Simulation and application to Europa. Icarus.

[27] Hesse M.A., Jordan J.S., Vance S.D., Oza A.V. (2022). Downward Oxidant Transport Through Europa’s Ice Shell by Density-Driven Brine Percolation. Geophys. Res. Lett..

[28] Soderlund K.M. (2019). Ocean Dynamics of Outer Solar System Satellites. Geophys. Res. Lett..

[29] Roukaerts A., Deman F., Van der Linden F., Carnat G., Bratkic A., Moreau S., Fripiat F. (2021). The biogeochemical role of a microbial biofilm in sea ice: Antarctic landfast sea ice as a case study. Elem. Sci. Anth..

[30] Showalter G.M. (2020). Acquisition, Degradation, and Cycling of Organic Matter within Sea-Ice Brines by Bacteria and Their Viruses.

[31] Soderlund K.M., Schmidt B.E., Wicht J., Blankenship D.D. (2013). Ocean-driven heating of Europa’s icy shell at low latitudes. Nat. Geosci..

[32] Zhu P., Manucharyan G.E., Thompson A.F., Goodman J.C., Vance S.D. (2017). The influence of meridional ice transport on Europa’s ocean stratification and heat content. Geophys. Res. Lett..

[33] Delgado J. (2007). Molecular Diffusion Coefficients of Organic Compounds in Water at Different Temperatures. J. Phase Equilibria Diffus..

[34] Kalousová K., Souček O., Tobie G., Choblet G., Čadek O. (2014). Ice melting and downward transport of meltwater by two-phase flow in Europa’s ice shell. J. Geophys. Res. Planets.

[35] Nguyen D., Maranger R. (2011). Respiration and bacterial carbon dynamics in Arctic Sea ice. Polar. Biol..

[36] Melosh H.J., Ekholm A.G., Showman A.P., Lorenz R.D. (2004). The temperature of Europa’s subsurface water ocean. Icarus.

[37] Servais P., Billen G., Rego J.V. (1985). Rate of Bacterial Mortality in Aquatic Environments. Appl. Environ. Microbiol..

[38] Marx J.G., Carpenter S.D., Deming J.W. (2009). Production of cryoprotectant extracellular polysaccharide substances (EPS) by the marine psychrophilic bacterium *Colwellia psychrerythraea* strain 34H under extreme conditions. Can. J. Microbiol..

[39] Jover L.F., Effler T.C., Buchan A., Wilhelm S.W., Weitz J.S. (2014). The elemental composition of virus particles: Implications for marine biogeochemical cycles. Nat. Rev. Microbiol..

[40] Dell’Anno A., Corinaldesi C., Danovaro R. (2015). Virus decomposition provides an important contribution to benthic deep-sea ecosystem functioning. Proc. Natl. Acad. Sci. USA.

[41] Vaqué D., Lara E., Arrieta J., Holding J.M., Sà E.L., Hendriks I.E., Coello-Camba A., Álvarez M., Agusti S., Wassmann P.F. (2019). Warming and CO_2_ Enhance Arctic Heterotrophic Microbial Activity. Front. Microbiol..

[42] Suttle C.A. (2007). Marine Viruses—Major Players in the Global Ecosystem. Nat. Rev. Microbiol..

[43] Lionard M., Péquin B., Lovejoy C., Vincent W.F. (2012). Benthic Cyanobacterial Mats in the High Arctic: Multi-Layer Structure and Fluorescence Responses to Osmotic Stress. Front. Microbiol..

[44] Mohit V., Culley A., Lovejoy C., Bouchard F., Vincent W.F. (2017). Hidden biofilms in a far northern lake and implications for the changing Arctic. NPJ Biofilms Microbiomes.

[45] Murga R., Stewart P.S., Daly D. (1995). Quantitative analysis of biofilm thickness variability. Biotechnol. Bioeng..

[46] Piculell M., Welander P., Jönsson K., Welander T. (2015). Evaluating the effect of biofilm thickness on nitrification in moving bed biofilm reactors. Environ. Technol..

[47] Suarez C., Piculell M., Modin O., Langenheder S., Persson F., Hermansson M. (2019). Thickness determines microbial community structure and function in nitrifying biofilms via deterministic assembly. Sci. Rep..

[48] Bartlett S., Wong M.L. (2020). Defining Lyfe in the Universe: From Three Privileged Functions to Four Pillars. Life.

[49] Waite J.H., Glein C.R., Perryman R.S., Teolis B.D., Magee B.A., Miller G., Grimes J., Perry M.E., Miller K.E., Bouquet A. (2017). Cassini finds molecular hydrogen in the Enceladus plume: Evidence for hydrothermal processes. Science.

[50] Smith S.V. (1984). Phosphorus versus nitrogen limitation in the marine environment1. Limnol. Oceanogr..

[51] Herbert R.A. (1999). Nitrogen cycling in coastal marine ecosystems. FEMS Microbiol. Rev..

[52] Pham A.L.D. (2019). Understanding Ocean Iron Dynamics and Impacts on Marine Ecosystems. Ph.D. Thesis.

[53] Beckett S.J., Williams H. (2013). Coevolutionary diversification creates nested-modular structure in phage–bacteria interaction networks. Interface Focus.

